# “The Flow in the Funnel”: Modeling Organizational and Individual Decision-Making for Designing Financial AI-Based Systems

**DOI:** 10.3389/fpsyg.2021.697101

**Published:** 2021-07-26

**Authors:** Alessandra Talamo, Silvia Marocco, Chiara Tricol

**Affiliations:** Department of Social and Developmental Psychology, Sapienza University of Rome, Rome, Italy

**Keywords:** decision-making, financial intelligence, artificial intelligence, qualitative methods, organizational psychology, social ergonomics

## Abstract

Nowadays, the current application of artificial intelligence (AI) to financial context is opening a new field of study, named financial intelligence, in which the implementation of AI-based solutions as “financial brain” aims at assisting in complex decision-making (DM) processes as wealth and risk management, financial security, financial consulting, and blockchain. For venture capitalist organizations (VCOs), this aspect becomes even more critical, since different actors (shareholders, bondholders, management, suppliers, customers) with different DM behaviors are involved. One last layer of complexity is the potential variation of behaviors performed by managers even in presence of fixed organizational goals. The aim of this study is twofold: a general analysis of the debate on implementing AI in DM processes is introduced, and a proposal for modeling financial AI-based services is presented. A set of qualitative methods based on the application of cultural psychology is presented for modeling financial DM processes of all actors involved in the process, machines as well as individuals and organizations. The integration of some design thinking techniques with strategic organizational counseling supports the modeling of a hierarchy of selective criteria of fund-seekers and the creation of an innovative value proposition accordingly with goals of VCOs to be represented and supported in AI-based systems. Implications suggest that human/AI integration in the field can be implemented by developing systems where AI can be conceived in two distinct functions: (a) automation: treating Big Data from the market defined by management of VCO; and (b) support: creating alert systems that are coherent with ordered weighted decisional criteria of VCO.

## Introduction

Artificial intelligence applied to decision-making (DM) processes has already been implemented in many fields (Galiano et al., 2019; Triberti et al., 2020; Bayrak et al., 2021) where it proves to have great potential. The current application of artificial intelligence (AI) to finance is nowadays opening a new field of study, named *financial intelligence*, in which the implementation of AI-based solutions as a “financial brain” aims at assisting in complex DM processes as wealth and risk management, financial security, financial consulting, and blockchain (Zheng et al., 2019).

Making investment decisions is usually considered a challenging task for investors, because it is a process based on risky, complex, and consequential choices (Shanmuganathan, 2020) on which trust both funding organizations and fund-seekers should rely. Furthermore, investment decisions are frequently influenced by emotional and cognitive biases, such as, overconfidence, and limited cognitive abilities. If individual DM is already challenging, at the layer of organizational contexts, such as venture capitalist organizations (VCOs), this aspect becomes even more critical, since different actors (shareholders, bondholders, management, suppliers, customers) with varying behaviors of DM are involved (De Bondt and Thaler, 1995). One last layer of complexity is the potential variation of behaviors performed by managers even in the presence of fixed organizational goals (Socea, 2012).

AI Contribution To The Field can be implemented in several aspects of the financial DM process, such as information collection and analysis, standardization of criteria of investments, and automation of customer interaction services. Nevertheless, recent findings show that the acceptance of AI-based solutions in DM by management is still an open issue within financial organizations since attitudes of manager toward intelligent agents are still unbalanced regarding human intervention in DM (Haesevoets et al., 2021).

Within this scenario, a core role can be played by tools that support AI modeling in designing financial AI-based solutions, which blend human/machine contribution in DM in the emerging field of explainable financial AI. In these AI-based solutions, the process on which results rely is transparent and understandable to users. What follows is an analysis of the debate on implementing AI in DM processes is introduced, and a proposal for modeling financial AI-based services is presented.

## AI Role in DM: State of the Art

Although the potential impact of AI in DM is proved to be significant (Zheng et al., 2019; Lepri et al., 2021), it led many practitioners and researchers in the field to take divergent points of view (Duan et al., 2019). The debate on human/technology relationships, even in AI, is not new: since the end of the last century, prominent scholars in the field have started positioning on contrasting perspectives, so that we can distinguish *techno-enthusiasts*, the true believers and supporters of technology and post-humanity, and *techno-skeptics*, who are more cautious and critical about future AI implementation in DM. These two divergent positions can be differentiated by focusing on specific issues:

### Objectivity of AI vs. Subjectivity of Human Beings

On one hand, techno-enthusiasts believe that the objectivity conferred by technology is an added value because it reduces the variability of human error. Specifically, they argue that algorithmic DM processes can lead to more objective decisions than those made by humans, which may be influenced by individual bias, conflicts of interest, or fatigue (Lepri et al., 2021). On the other hand, techno-skeptics firmly state that machines can only partially simulate but never duplicate the unique mental life of humans; in fact, machines cannot feel or understand the complexity of real-life situations (Postman, 1993). Furthermore, in this perspective, the objectivity of AI and other intelligent technologies fails in making decisions with uncertain circumstances. Although AI systems can assist human decision-makers with predictive analytics, they are less capable of understanding common-sense situations (Guszcza et al., 2017) and unpredictable environments, particularly outside of a predefined domain of knowledge (Brynjolfsson and McAfee, 2012).

### The Lack of Transparency of AI

One of the main concerns of techno-skeptics is the lack of transparency of AI. In this regard, most skeptics criticize algorithmic DM processes for the threat of privacy invasion, information asymmetry, and discrimination (Lepri et al., 2021). Moreover, AI and algorithmic DM processes are increasingly challenged for their black-box nature: although AI systems enable robust predictions, most users have little awareness and knowledge of how these systems make decisions. Hence, the lack of transparency hinders comprehension and negatively affects trust (Shin, 2021). This issue has fostered a new research field on explainable AI (XAI), which aims to substantially improve the trust and transparency of AI-based systems (Adadi and Berrada, 2018).

### Augmentation vs. Automation

On one side, techno-enthusiasts aim at demonstrating the use of AI software systems and machines for automating tasks to eliminate human input. On the other, techno-skeptics are becoming more apprehensive, fearing that intelligent machines may soon take them over. In this regard, Stephen Hawking has noted that “the development of full artificial intelligence could spell the end of the human race” (Cellan-Jones, 2014), and Bill Gates has also stressed that humans should be concerned about the threat caused by AI (Rawlinson, 2015; Duan et al., 2019). As a result, some researchers have reframed the threat of automation as an opportunity for augmentation, proving that augmented intelligence can supplement and amplify human capabilities for cognition and collaboration (Miller, 2018).

Despite the fear and skepticism of some scholars, it is evident that the potential of AI implementation cannot be denied. According to the vision of some AI practitioners and researchers, it seems more meaningful to see AI in DM processes as an augmentation tool, able to extend human capabilities and judgments, rather than as automation, able to replace them (Miller, 2018; Wilson and Daugherty, 2018; Duan et al., 2019). In line with this last position, Jarrahi asserts that “artificial intelligence systems should be designed with the intention of augmenting, not replacing, human contributions” (Jarrahi, 2018, p. 584).

Within this debate, a question arises: how can humans and AI act in a complementary way in DM? The position starts from a specific perspective on innovation in technological advances. In the 1970s, Thierry Gaudin (1978) developed a human-centered theory of innovation that may help us in reasoning in a practical way on the human–technology relationship. In the proposal of Gaudin, it is not just the technological development that promotes or inhibits innovation processes, but rather the behavior of organizations, considered as vital beings, with their missions, their evolutionary paths, and their modes of functioning (Talamo et al., 2016).

## From Human/Machine Interaction to Human/AI Integration: A Psychological Perspective

Since the 1980s, a growing body of literature on human/machine interaction has produced consolidated evidence on the “external side” of user experience, that is, the front-end layer of interacting with systems. However, the fast development of AI implementation pushes us to reason on different layers, focusing on automation and replication of contextualized human reasoning models to shape the “internal side of technologies.”

In the last 20 years, research on organizational disasters has already demonstrated the risk of taking an ingenuous perspective on technology implementation where technical, rationale, automatic, and general were considered preferable to practical, socialized, and contingent (Heath and Luff, 2000). Additionally, some highlighted the crucial role of proper treatment of information to support organizations and individuals in avoiding organizational disasters due to mistakes in information management in personal and collective DM processes (Choo, 2008). There is also growing evidence of the relevance of including ecological criteria for designing technologies (Talamo et al., 2011, 2015, 2017; Giorgi et al., 2013), to capture the complexity and contingency of real-life actions in specific situations.

Therefore, research on human/AI integration could benefit by considering some reflections from Cultural Psychology and more specifically from scholars by Activity Theory (AT) (Leont’ev, 1974, 1978; Engeström, 1987, 2000) who focus on three central concepts in analyzing the relationship between persons and technologies:

### An Asymmetrical Interaction Between the Subject and the Object

Activity theory conceives human activity as a form of doing, performed by a subject and directed to an object, whose outcome will satisfy the needs of the subject. This interaction between the subject and the object is not a symmetrical relationship between two components of a system, since it is initiated and executed by the subject to meet its needs (Pickering, 1993, 1995). AI, for example, follows a program written by an IT developer who wants to respond to a need: technology, in fact, only has “the ability to act but not the need to act” (Kaptelinin and Nardi, 2006, p.33).

### Intentionality of Human Beings

Agency, “the ability to act in the sense of producing effects according to an intention” (Kaptelinin and Nardi, 2006, p.33), is another crucial concept covered by socio-cognitive theories. For Leont’ev (1974, 1978), the primary type of agency is that of individual human subjects because it is closely related to the concept of human intentionality (Stetsenko and Arievitch, 2004). According to AT, intentionality is considered as a property of sole individual subjects. As Rose et al. (2005) observed, humans have “self-awareness, social awareness, interpretation, intentionality, and the attribution of agency to others,” which are not available to non-living things, such as technological systems.

### Mediation of Tools

Finally, the above-mentioned asymmetrical interaction between the subject and the object can be mediated by a tool, a physical artifact, or an intangible tool (e.g., ideas and procedures), which allows the subject to reach the final goal (Leont’ev, 1974, 1978). For example, technological tools, as activity mediators, can facilitate the interaction that allows the subject to achieve his goals, but they can also create boundaries because of the way in which the technology is implemented in those specific tools (Kuutti, 1996). Mediation of tools can also support the creation of interobjectivity among team members (Talamo and Pozzi, 2011).

## Preceding AI Development in Financial Systems: Modeling and Integrating Multi-Actor DM Processes

As previously illustrated, while the contribution of AI in the financial sector requires the automation of DM processes to collect and analyze information, standardize investment criteria, and automate customer interaction services, Cultural Psychology ascribes to humans the primacy in DM processes, supporting the intentionality of human beings and the mediation role of tools. Hence, in order to enable functional human/AI integration and to guarantee proper functioning of AI-based systems, it is necessary to study in depth the DM model in the field.

Considering this, we propose a possible set of qualitative tools for the design of an AI-based financial DM support system. The tools we chose can be divided into two categories according to their objectives:

**Tools to produce knowledge** (e.g., narrative interviews, maieutic clinical dialogs): to fully understand DM processes of all the actors involved in the financial field before AI implementation.
**Tools to model processes** (e.g., user journey map, activity diagram): to model both DM processes of the provider and the user in order to create a bridge that can offer efficacy and efficiency to the provider and satisfaction of needs to the users.

To show how these qualitative tools can help in modeling DM processes, we refer to a case study on which we applied the methods for the design of an AI platform to support decision-makers from a VCO. The original explicit request by VCO managers was to help information technology (IT) developers in modeling the AI system by tracing management DM, focusing on a specific DM step: comprehend which criteria and sub-criteria would “filter” the fund-seekers into the funnel flow in order to be judged for funding opportunities. The main issue was to figure out which aspects of human DM processes could be automated, and which human DM processes could be supported but not substituted.

To this aim, on one hand, we explored expected goals of VCO enhancing its awareness; on the other, we studied the fund-seekers to model their decision flow.

### Enhancing VCO Awareness: Strategic Organizational Counseling

In most cases, when dealing with introducing AI in financial DM, attention is placed on modeling individual DM processes to be replicated and automated.

Nevertheless, we faced a lack of methods for modeling organizational DM in the financial field, not just in terms of formal declaration, rather with a necessary focus on shared implicit managerial criteria for providing funding. The method we implemented, SOC, consists of dialogical sessions with different managers guided by a psychologist implementing maieutic clinical techniques oriented to make implicit criteria arise in explicit talk. This tool made it possible to:

Identify and model DM processes at an organizational layer (transversal to different managers).
Differentiate the potential value proposition by the organization to different target among fund-seekers.
Model scouting criteria on fund-seekers to orient financial decisions.

The result of this activity is twofold: on one side, it produced increased awareness in management on the complexity of DM processes they have to deal with and highlighted the need to share even implicit criteria used by different managers in different contextual circumstances; on the other, it produced descriptive charts of DM flows to be implemented in the platform. This process made it possible to align the system development team by highlighting two distinct roles of AI as potential support of the organizational DM: first, AI would automatically analyze Big Data from the market to suggest which trends should be preferentially funded; and second, AI could support managers in DM by signaling which inputs by fund-seekers would fit better with goals of the VCO. From this process, it was possible to shape ordered weighted averaging (OWA) operators (Merigò and Gil-Lafuente, 2010), to support VCO defining cases in which fund-seekers fit with funding criteria.

### Exploring Decision Flow of Fund-Seekers

As Suchman (1987) demonstrated, human behavior in complex DM tasks is often complicated and can sometimes look chaotic. Nevertheless, once we observe it using descriptive tools from anthropology and psychology, we find more easily rationales that explain those behaviors in terms of personal and organizational contextualized objectives that actors are pursuing. For these reasons, design and usability practitioners elaborated over time different sets of qualitative methods for collecting valuable data on user behaviors (Cooper et al., 2007; Talamo et al., 2016; Recupero et al., 2018). The method we chose for the user research is the narrative interviews (Atkinson, 2002). This tool allowed us to collect perspectives of fund-seekers and the meanings they attribute to different steps of financial decision flow in terms of feelings, cognitions, representations of gain, and pain.

### Modeling Activities of Fund-Seekers and DM Processes

**FIGURE 1:**
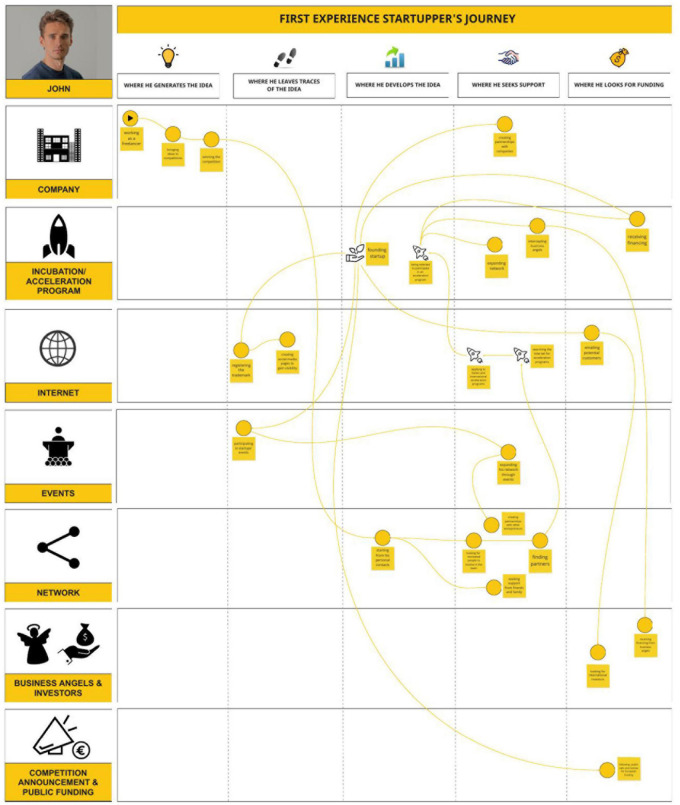
User journey map.

Data collected were then employed to model activities of fund-seekers and DM processes. The tools we used belong to the Design Thinking approach, and some of these have been customized *ad hoc* to meet the scope of the VCO management. A tool that proved to be crucial in this process was the user journey map (Stickdorn and Schneider, 2011). This tool, configured as an oriented graph, provides a vivid, concise, structured, and precise visualization of the user experience, according to the decision flow of fund-seekers. It also enabled IT developers to understand the contexts and channels through which the platform could intercept fund-seekers and the moments and the kind of operations in which AI contribution could be most appropriate (see Figure 1).

### Bridging Funders and Fund-Seekers

**FIGURE 2:**
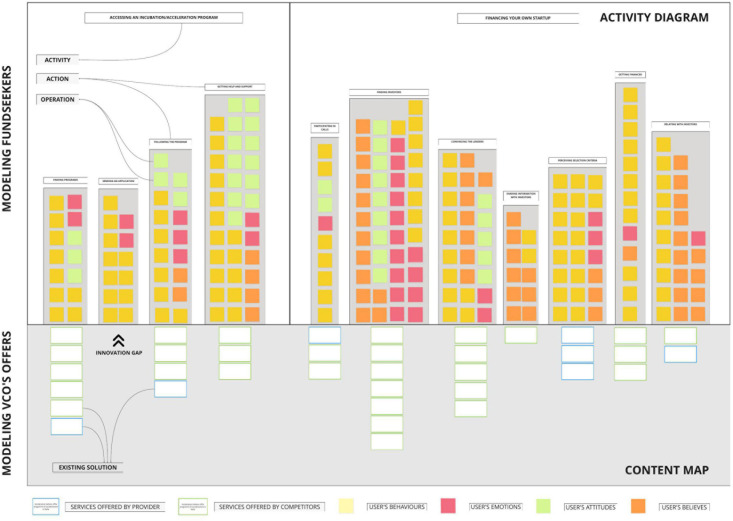
Structure of the activity diagram.

A crucial tool for creating a bridge between DM processes of fund-seekers and VCO was the activity diagram (Young, 2008). Consistently with AT, this tool, by structuring *activities, actions*, and *operations* of fund-seekers (Kaptelinin and Nardi, 2006) and matching them with services of VCO, proved to be very useful in identifying problems, developing potential bridging solutions, and recognizing spaces for innovation to create *ad hoc* AI-supported services (see Figure 2).

As shown in Figure 2, the critical added value of modeling prospective users in the activity diagram relies on the potential comparison that the tool offers to match existing solutions (by the funding organization and its competitors). The content map section of this tool indicates some innovation gaps between actual financial services and activities and needs of fund-seekers, which are still not satisfied. Therefore, this modeling method can foster in funding organizations the capacity of creating innovative services to be implemented in AI-based systems.

## Discussion

Since DM in the financial field is a complex multilayer and multi-actor process, we propose a specific sequence of action-research activities aimed at modeling specific phases of DM processes by different actors:

**Enhancing organization’s DM awareness:** This step aimed at producing an increased awareness in management on their own intentions and funding criteria to finalize the different ways AI will support their decision-making.
**Exploring fund-seekers:** This step aimed at studying the potential fund-seekers and their psychological world to collect data on which the modeling activity can be based.
**Modeling activities of fund-seekers and DM processes:** This step led to a full-fledged view of the fund-seekers. The collected data were beneficial to the developers of systems and the VCO, providing insights about the contexts and the channels through which the platform could intercept fund-seekers.
**Bridging funders and fund-seekers:** This last step, matching DM flow of fund-seekers with services of VCO, proved to be very useful in identifying problems, developing potential bridging solutions, and recognizing new spaces for innovation.

As the financial field is a promising multi-actor research area, the contribution of sociocultural approach from psychology and proposed methods can play a crucial role. In fact, Design Thinking combined with maieutic techniques, typical of expertise of psychologists, fosters modeling the complexity of DM systems emerging from different actors around funding decisions. Within the development team, the psychologist then becomes a mediator between IT developers and the VCO for which the system is developed.

Implications of this case study suggest that human/AI integration in the financial field can be successfully implemented by developing systems where AI can be conceived in two distinct functions: (a) automation/augmentation: treating Big Data from the market defined by VCO management; and (b) human/AI integration: creating OWA-based alert systems that support managers in taking decisions coherently with criteria of VCO.

Finally, we argue that, to achieve effective results in the design of complex IT systems that use AI in DM, technology development, albeit providing an enormous contribution, cannot disregard a deep comprehension of real practices by human actors. Therefore, as Kelly (2012) says: “*This is not a race against machines this is a race with machines.*”

## Data Availability Statement

The raw data supporting the conclusions of this article will be made available by the authors, without undue reservation.

## Ethics Statement

Ethical review and approval was not required for the study on human participants in accordance with the local legislation and institutional requirements. The patients/participants provided their written informed consent to participate in this study.

## Author Contributions

AT conceptualized the ideas presented in the article, defined the theoretical framework, and supervised the whole process. SM and CT wrote a first draft and helped to edit the manuscript. All authors contributed to revision, read, and approved the submitted version.

## Conflict of Interest

This article is based on a use case which has been part of a broader project based on the idea of Archangel AdVenture, a seed capital investment firm based in Italy (https://www.archangeladventure.it), of adopting AI to leverage open-source intelligence techniques applied to technology scouting for the purpose of investing in new ventures. The authors were involved by Teleconsys as experts in User-centered design research and were free to decide how to conduct the research. Some key persons from funders’ organizations were interviewed (as described in the paper) during the SOC collection of data as research subjects. The design of research procedures and tools, data analysis and interpretation, and the writing of this article were made by the authors. The authors declare to have informed the funders about the decision to submit the article for scientific publication.

## Publisher’s Note

All claims expressed in this article are solely those of the authors and do not necessarily represent those of their affiliated organizations, or those of the publisher, the editors and the reviewers. Any product that may be evaluated in this article, or claim that may be made by its manufacturer, is not guaranteed or endorsed by the publisher.
